# Supplementary material for: Angular and linear speed cells in the parahippocampal circuits
Source: Nat Commun. 2022 Apr 7;13:1907. doi: 10.1038/s41467-022-29583-z (PMC8991198; doi:10.1038/s41467-022-29583-z)
Supplement: Supplementary file 1 — Supplementary Information [file 41467_2022_29583_MOESM1_ESM.pdf]

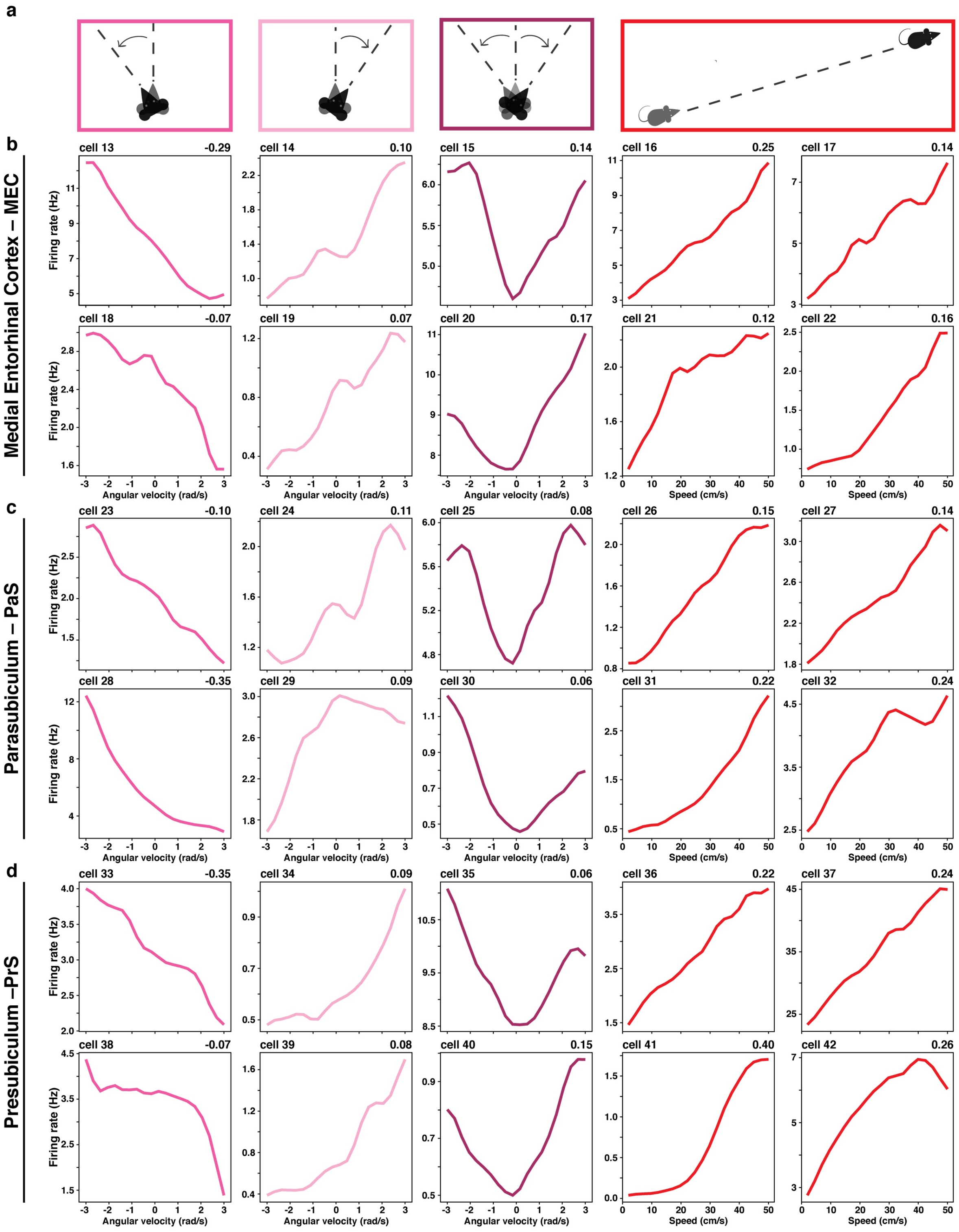

Supplementary Fig. 1 Extended examples of speed and Angular head velocity cells

(**a**) Schematic representation of three type of angular head velocity (AHV) movement and linear speed, from left to right: counterclockwise (CCW, dark pink), clockwise (CW, light pink), bidirectional (BiDir, purple) and linear speed (red). 30 additional examples of self-motion cells: 6 AHV and 4 speed cells in each region; medial entorhinal cortex (**b**),

parasubiculum (**c**) and presubiculum (**d**). The firing rate is represented as a function of angular velocity (in rad/s) or speed (in cm/s). AHV or speed scores are reported in the upper right corner. Cell ID are reported in the upper left corner. From left to right: CCW-AHV (dark pink), CW-AHV (light pink), BiDir-AHV (purple) and speed (red, last two columns). Note that the high values of the rate of cell 37 suggest that it may be an interneuron. Credits to Silvia Girardi for schematics and drawings.

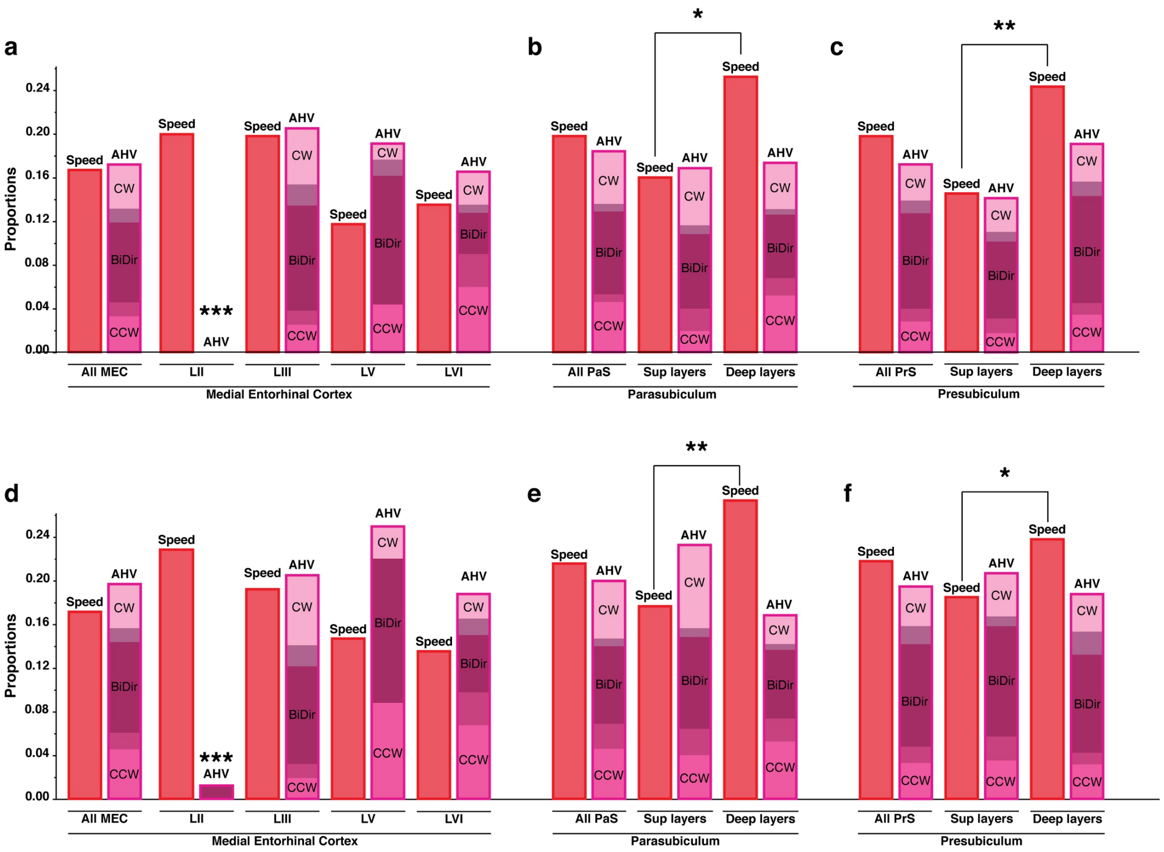

Supplementary Fig. 2 Distribution of angular head velocity and linear speed modulation across parahippocampal layer (single-cell and region-wise shuffling method)

Proportion of speed (red) and AHV cells (CW light pink, CCW dark pink, BiDir purple) across layers. Shaded areas represent overlap between CW (CCW) and BiDir cells. (**a–c**) Region-wise shuffling method: (**a**) Proportions in medial entorhinal cortex. Stars denote the significant absence of AHV cells in MEC LII (proportion z-test, pvalue <0.001). (**b**) Proportions in the parasubiculum. (**c**) Proportions in the presubiculum. (**d–f**) Single-cell shuffling method: Proportions in medial entorhinal cortex (**d**), in the parasubiculum (**e**) and in the presubiculum (**f**). All tests are two sided. Stars denote the significant difference in speed cells between superficial and deep layers both in PrS and PaS (t-test, *** pvalue <0.001, ** pvalue <0.01 and * pvalue <0.05 respectively). Cells identified with the two methods significantly overlapped (about 90% of the cells, Binomial test pvalue <0.001).

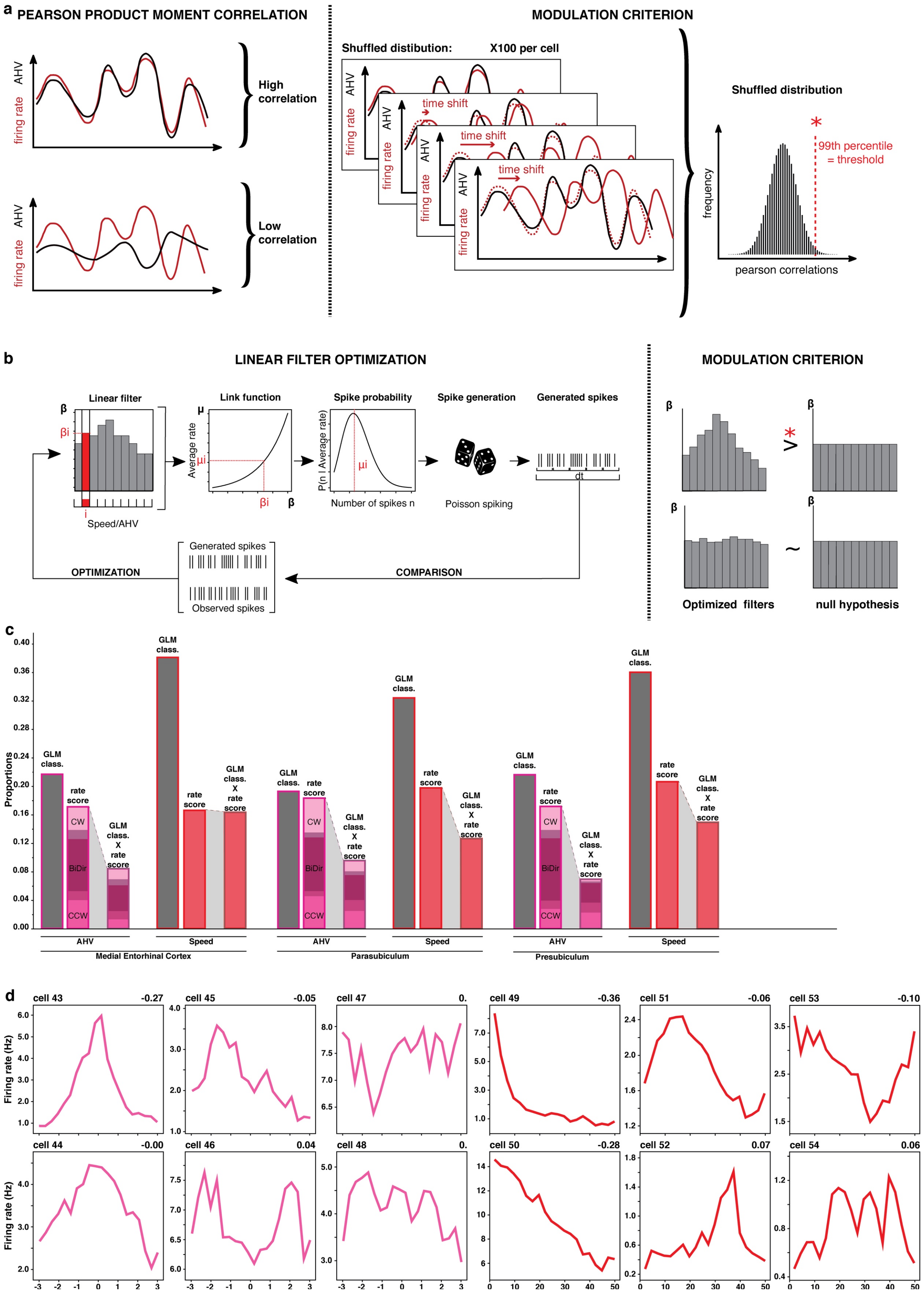

Supplementary Fig. 3 Comparison between scoring methods: correlation vs. GLM

(**a**) Schematics of the correlation method. The session-wide Pearson correlation between the firing rate of the cell (red) and the angular head velocity (black) is computed. Two examples of high (left, top) and low (left, bottom) correlations are illustrated. Significance is calculated with a shuffling procedure (right) in which the firing rate is shifted in time of a random amount, 100 times for each cell and a shuffled score is calculated. Shuffled scores of all cells in the same region are pooled together to obtain a null distribution of score. Cells are labelled as AHV modulated if their score exceed the 99th percentile of the null distribution (rightmost part). (**b**) Schematics of the generalized linear model (GLM) method. First the weights β of the GLM model are optimized with a maximum likelihood procedure (left). The value β_i corresponding to the bin i of the instantaneous angular head velocity determines the value μ_i of the instantaneous firing rate through an exponential link function. The value μ_i is the average of the Poisson distribution giving the probability $P(n|\mu_i)$ to observe a certain number of spikes n in the time interval dt at which the AHV signal is sampled (20 ms in our case). This distribution is used to generate, through a Poisson spiking process, a train of spikes to be compared with the ones experimentally observed. The weights are adjusted to maximize the similarity across the whole session. The cell is labelled as modulated if the likelihood of the optimized model is significantly larger than the null model with constant β fixed at the average firing rate of the cell (right) (**c**) Intersection between correlation and GLM modulated cells in MEC (GLM modulated AHV cells: 21.7%, n=86; correlation modulated AHV cells: 16.9%, n=67; intersection: 9.6%, n=38 | GLM modulated speed cells: 38.1%, n=151; correlation modulated speed cells: 16.7%, n=66; intersection: 12.6%, n=50), parasubiculum (GLM modulated AHV cells: 19.3%, n=84; correlation modulated AHV cells: 17.2%, n=75; intersection: 6.9%, n=30 | GLM modulated speed cells: 32.4%, n=141; correlation modulated speed cells: 19.8%, n=86; intersection: 14.9%, n=65), and presubiculum (GLM modulated AHV cells: 21.6%, n=131; correlation modulated AHV cells: 17.2%, n=104; intersection: 8.4%, n=51 | GLM modulated speed cells: 36%, n=218; correlation modulated speed cells: 20.7%, n=125; intersection: 16.4%, n=99). The intersection between correlation modulated and GLM modulated cells is in each case significantly larger than expected by chance (2-sided binomial test, pvalue <0.001). (**d**) Example of self-motion cells only selected by the GLM method. Left panel (pink traces, cells 43–48): AHV cells. Right panel (red traces, cells 49–54): speed cells. For each panel: the two first cells on the left are anticorrelated (maximum firing a low speed, cells 43–44 and 49–50), the two next cells in the middle are coding for a specific speed (cells 45–46 and 51–52) and the two last cells on the right are showing irregular profiles (cells 47–48 and 53–54, cells

47 and 53 seems to be anticorrelated for a specific speed band). Note that both AHV and speed sigmoid coding (presented in SF4) are picked up by Pearson method.

Comment on scoring method (supplementary discussion): The Pearson correlation method and the GLM approach have complementary advantages and weaknesses. The Pearson method assumes that the modulation of the firing rate is linear or close to linear but that it does not require any further arbitrariness in the fixing of additional parameters. Similar correlative methods associated with a region-wise or single-cell shuffling have been used in many previous coding studies, including the one evidencing the existence of speed cells in MEC[17]. The GLM approach can detect more general forms of modulation but is subject to choices (e.g., binning size, regularization procedure, form of the link function) that are, at least to some degree, arbitrary. This approach was recently used to evidence a few conjunctive AHV cells in the MEC[21].

We have analysed our dataset using these two approaches – estimating that both cross-validation (Pearson) and bootstrapping (GLM) are effective methods for building statistical null models. We observed that the GLM analysis yielded a larger percentage of modulated cells – yet the majority was overlapping with the population detected by the Pearson method. Cells solely detected by the GLM approach showed either low modulation, non-linear tuning curves, or non-recognizable shape in their tuning curves. An interesting case is the one of anti-correlated self-motion cell, which constituted a large percentage of the cell detected only by GLM methods. These cells suffer from the confounding factor that their activity could be more related to any process that takes place in the absence of movement, than with movement itself. A carefully controlled experimental setting would be needed to disentangle these two aspects. Past studies have often made the choice not to report anticorrelation when qualifying other spatial and directional component (i.e., grid, place, and HD cells). Regardless, the main results put forward in our study held true independently of the method used. For sake of clarity, we opted to report in the main figures only the results on cells selected by the Pearson method. However, any differences with the GLM selected cells are highlighted in the main text. Note that the two population overlap greatly. We therefore also report results with cells only selected with the GLM and not by the Pearson – "GLM only".

We privilege the Pearson approach in the main figure because it is non-parametric, have low variance and does not rely in hyperparameter tuning. This means that: (i) Combined with a

region-wise (or single-cell) shuffle, it affords a very large statistical power: pooling the number of shuffles allows to have a statistical null distribution with hundreds of thousands of data points. Such a method was very conservative and yielded the lower percentages of modulated cells. In our opinion, the very low rate of false positives guaranteed by this method is a strength in the interpretation of the significance and reproducibility of the results. (ii) The Pearson method has a lower variance than GLM – its hypothesis class is much smaller than the one of GLMs – preventing overfitting. (iii) This method is also non-parametric: it does not require a choice of bins and bin size and no choice of regularization hyperparameters and procedure. This makes the results more easily interpretable, reproducible and generalizable. (iv) Our analysis yields a direct measure of explained variance in the Pearson score, whose absolute value is directly interpretable as the fraction of variability of the firing that is explained by the correlate. We check variable covariation by looking at the correlation of different score, which we find to be absent of or mild.

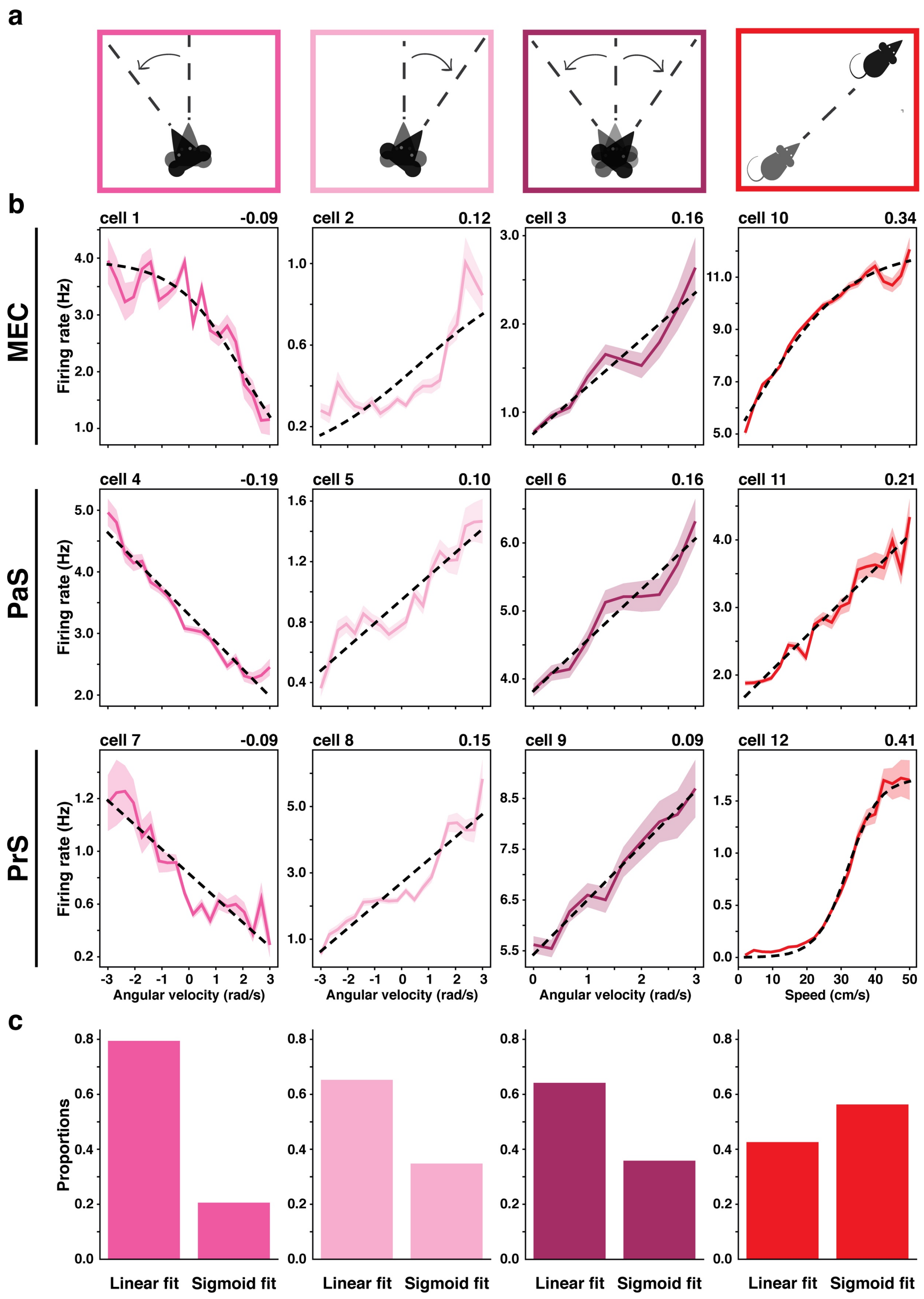

Supplementary Fig. 4 Linear and sigmoidal fit in angular head velocity (AHV) and speed modulated cells

(**a**) Schematic representation of the behavioural correlate modulating the rate. From left to right: AHV CCW (dark pink), AHV CW (light pink), AHV BiDir (purple), linear speed (red).

(**b**) Examples of tuning curves of cells in MEC (top row), PaS (middle row) and PrS (bottom row), columns colour coded and arranged as in (a). Solid lines represent the average firing rate at a given value of AHV (speed) across the recording session, shaded areas represent the standard error of the mean and dotted line the best linear (sigmoidal) fit. Cell scores are reported on the top right corner and cell ID in the top left corner. (**c**) Proportion of linear and sigmoidal cell in the total population. From left to right: AHV CCW (Linear: 79.4%; Sigmoidal: 20.6%), AHV CW (Linear: 65.2%; Sigmoidal: 34.8%), AHV BiDir (Linear: 64.1%; Sigmoidal: 35.9%), speed (Linear: 42.6%; Sigmoidal: 56.3%). Note that the sigmoidal fits are often quasi-linear. Credits to Silvia Girardi for schematics and drawings.

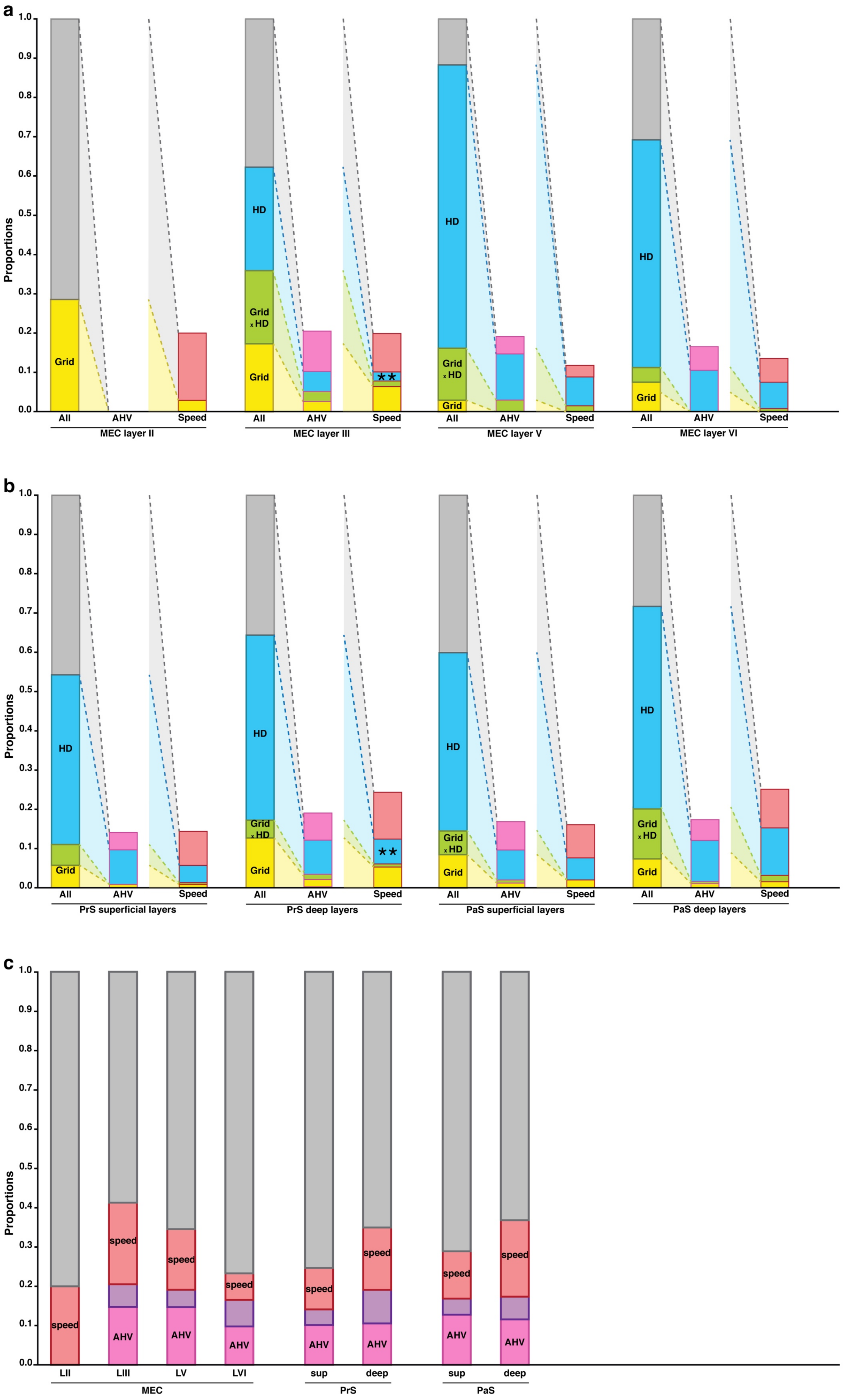
a
b
c
Proportions
1.0
0.9
0.8
0.7
0.6
0.5
0.4
0.3
0.2
0.1
0.0
Grid
Grid x HD
HD
**
All
AHV
Speed
MEC layer II
MEC layer III
MEC layer V
MEC layer VI
PrS superficial layers
PrS deep layers
PaS superficial layers
PaS deep layers
speed
AHV
LII
LIII
LV
LVI
sup
deep
MEC
PrS
PaS

Supplementary Fig. 5 Distribution of conjunctive coding across areas and layers

(**a–b**) Proportions of grid (yellow), HD (blue), grid x HD (green) cells in the whole layer population (left bar, black outline), within the AHV cell population (central bar, pink outline) and within speed cell population (right bar, red outline). Grey bars represent cells that are neither coding for grid nor HD. Pink bars in the AHV population histograms represents AHV cells that are neither coding for grid nor HD. Red bars in the speed population histograms represents speed cells that are neither coding for grid nor HD. All tests are two sided. Stars denote a significant change in proportions of a specific type of conjunctive cells within either the AHV or the speed population from what would be expected from the layer proportions within the general population. All proportions were as expected, except for an underrepresentation of speed X HD in MEC L III and in deep Prs (proportion z-test: *** pvalue <0.001, ** pvalue <0.01 and * pvalue <0.05 respectively). **MEC** (**a**): MEC layer II (grid: 28.6%; HD: 2%; grid x HD: 0%), layer III (grid: 35.9%; HD: 44.9%; grid x HD: 18.6%), layer V (grid: 16.2%; HD: 85.3%; grid x HD: 13.2%), and layer VI (grid: 11.3%; HD: 61.7%; grid x HD: 3.7%). **PrS** (**b**, left): PrS superficial layers (grid: 11%; HD: 48.5%; grid x HD: 5.3%), and PrS deep layers (grid: 17.2%; HD: 51.6%; grid x HD: 4.5%). **PaS** (**b**, right): PaS superficial layers grid: (14.5%; HD: 51.4%; grid x HD: 6%), PaS deep layers (grid:20%; HD: 64.2%; grid x HD: 12.6%). Note the quasi-absence of HD and AHV cells in MEC LII. (**c**) Pink and red bars here represent the whole population of AHV (pink) and speed (red) cells in a given layer. Note that those are different populations than in (a–b). Purple bars represent cells whose activity is conjunctively modulated by speed and by AHV. From left to right: MEC layer II (AHV: 0%, speed: 20%, AHV x speed: 0%), MEC layer III (AHV: 20.5%, speed: 19.9%, AHV x speed: 5.8%), MEC layer V (AHV: 19.1%, speed: 11.8%, AHV x speed: 4.4%), MEC layer VI (AHV: 16.5%, speed: 13.5%, AHV x speed: 6.8%), PrS superficial layers (AHV: 14.1%, speed: 14.5%, AHV x speed: 3.9%), PrS deep layers (AHV: 19.1%, speed: 24.3%, AHV x speed: 8.5%), PaS superficial layers (AHV: 16.9%, speed: 16.1%, AHV x speed: 4%) and PaS deep layers (AHV: 17.4%, speed: 25.3%, AHV x speed: 5.8%).

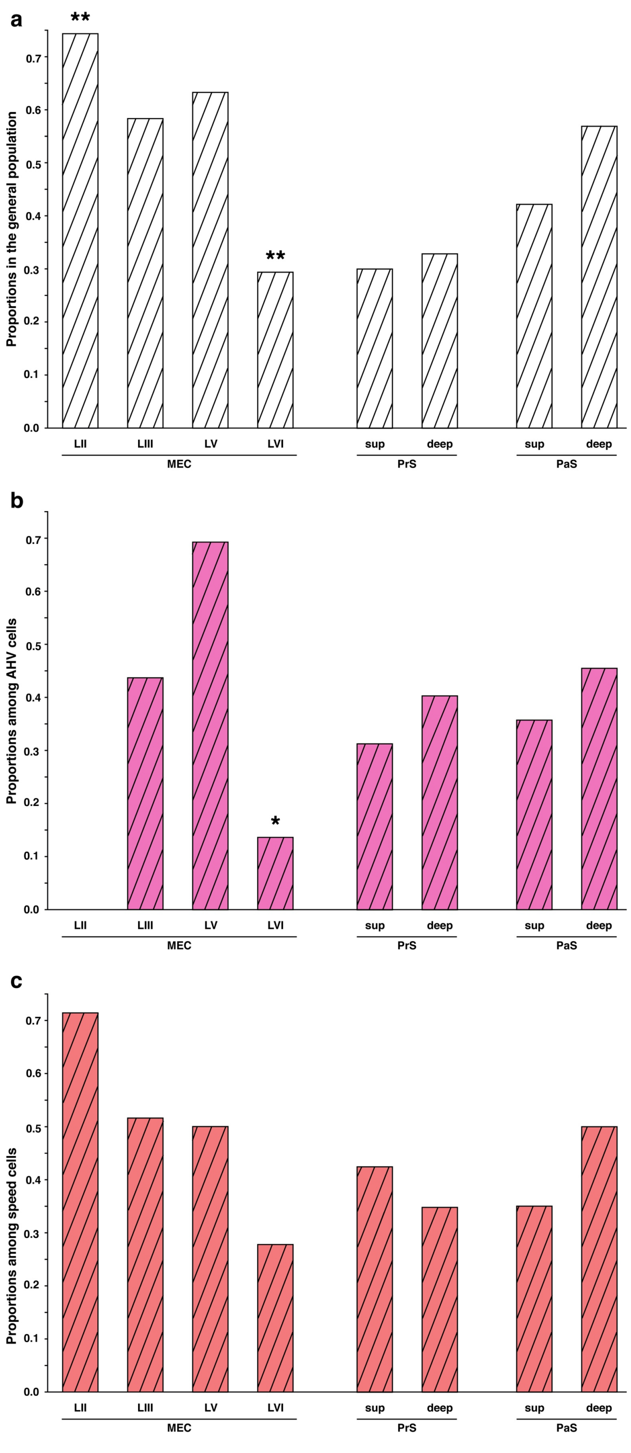

Supplementary Fig. 6 Distribution of theta modulation by layer

Dashed bars represent proportions of theta modulated cells for each category of cells considered. Cells were considered theta modulated when their mean power in a 2 Hz window centred in the peak in the 5- to 11-Hz frequency range was at least fivefold greater than the mean spectral power in the 0- to 125-Hz range. (**a**) Percentages of theta modulated cells in the whole population (white dashed bars). From left to right: MEC LII (74.3%), MEC LIII

(58.3%), MEC LV (63.2%), MEC LVI (29.3%), PrS superficial layers (29.9%), PrS deep layers (32.8%), PaS superficial layers (42.2%) and PaS deep layers (56.8%). Stars denote a significant difference in proportion of theta modulated cell in the layer considered, compared to the average theta modulation across all layers (proportion z-test, ** pvalue <0.01). (**b**) Percentages of theta modulated cells in the AHV population (pink dashed bars). From left to right: MEC LII (0%), MEC LIII (43.8%), MEC LV (69.2%), MEC LVI (13.6%), PrS superficial layers (31.3%), PrS deep layers (40.2%), PaS superficial layers (35.7%) and PaS deep layers (45.5%). Stars denote a significant difference in proportion of AHV theta modulated cell from what would be expected given the average theta modulation in the general population (proportion z-test, * pvalue <0.05). (**c**) Percentages of theta modulated cells in the speed population (red dashed bars). From left to right: MEC LII (71.4%), MEC LIII (51.6%), MEC LV (50%), MEC LVI (27.8%), PrS superficial layers (42.4%), PaS deep layers (34.7%), PaS superficial layers (35%) and PaS deep layers (50%). All tests are two sided (proportion z-test, *** pvalue <0.001, ** pvalue <0.01 and * pvalue <0.05 respectively).
